# Guidelines and recommendations for radiographer staffing from the EU-REST project

**DOI:** 10.1186/s13244-025-02105-3

**Published:** 2025-11-03

**Authors:** Jonathan P. McNulty, Francis Zarb, the European Federation of Radiographer Societies, the European Society of Radiology

**Affiliations:** 1 Radiography and Diagnostic Imaging, School of Medicine, University College Dublin, Dublin, Ireland; 2 Department of Radiography, Faculty of Health Sciences, University of Malta, Msida, Malta; 3 EU-REST Consortium, Vienna, Austria; 4 European Federation of Radiographer Societies, Cumiera, Portugal; 5 European Society of Radiology, Vienna, Austria; 6 Department of Radiology, University College Cork, Cork, Ireland; 7 Department of Diagnostic and Interventional Radiology, University Hospital Dubrava, University of Zagreb School of Medicine, Zagreb, Croatia; 8 Division of Cardiovascular and Interventional Radiology, Department of Biomedical Imaging and Image-Guided Therapy, Medical University of Vienna, Vienna, Austria; 9 Discipline of Radiation Therapy, School of Medicine, Trinity Centre for Health Sciences, St. James’ Hospital, Dublin, Ireland; 10 LaTIM, INSERM, UMR1101, University of Brest, Brest, France; 11 Department of Radiation Oncology, Ege University Faculty of Medicine, Izmir, Turkey; 12 Radiation Research Unit, IEO, European Institute of Oncology IRCCS, Milan, Italy; 13 Department of Nuclear Medicine, Cliniques Universitaires St-Luc and Institute for Experimental and Clinical Research (IREC), Université Catholique de Louvain, Brussels, Belgium; 14 Cap Clinic, Servei de Radiofísica i Radioprotecció, Hospital de la Santa Creu i Sant Pau, Barcelona, Spain; 15 Canarian Comprehensive Cancer Center, San Roque University Hospital, Las Palmas, Spain; 16 Fernando Pessoa Canarias University, Las Palmas, Spain; 17 Applied Radiation Therapy Trinity, Discipline of Radiation Therapy, Trinity College Dublin, Dublin, Ireland; 18 Trinity St. James’s Cancer Institute, Dublin, Ireland; 19 Polytechnic University of Coimbra, IPC-ESTESC—Coimbra Health School, Medical Imaging and Radiotherapy, Coimbra, Portugal; 20 H&TRC—Centro de Investigação em Saúde e Tecnologia, Lisbon, Portugal; 21 Budapesti Műszaki és Gazdaságtudományi Egyetem (BME), Budapest, Hungary; 22 National Institute of Oncology, Budapest, Hungary; 23 Department of Health Sciences, European University, Nicosia, Cyprus; 24 Hospital Clínico San Carlos, Universidad Complutense de Madrid, Madrid, Spain

**Keywords:** Radiographers, Recommendations, Safety, Staffing, Workforce planning

## Abstract

**Abstract:**

This article outlines the radiographer workforce and staffing recommendations developed by the European Commission-funded EU-REST (European Union Radiation, Education, Staffing and Training) project. Focusing on radiographers in medical imaging, nuclear medicine, and radiotherapy, the project identified gaps in workforce planning across EU member states. Evidence-based guidelines to harmonise radiographer staffing levels and improve safety and quality in medical settings are proposed. A structured, workload-based approach for radiographer staffing using the World Health Organization’s Workload Indicators of Staffing Need (WISN) method, offering a comprehensive framework to optimise workforce planning, is outlined. The study emphasises the critical role of radiographers, who are increasingly involved in advanced and extended roles, driven by technological advancements, demographic changes, and evolving healthcare demands. However, challenges in defining workforce requirements persist due to the lack of standardised methodologies, leading to fragmented staffing guidelines. National registries to track workforce data and the recognition of emerging roles for radiographers, such as clinical research radiographers, academics, and advanced practitioners, are also recommended. By addressing these emerging needs and incorporating them into workforce planning, the study aims to enhance professional development and improve patient outcomes across EU member states. General recommendations for all professional groups include the maintenance of a central registry for each professional group and for relevant equipment by each EU Member State.

**Critical relevance statement:**

The radiographer workforce/staffing guidelines and recommendations developed by the EU-REST project propose a standardised approach to calculate radiographer staffing levels and provide recommendations regarding new and emerging professional roles and the need for centralised workforce data registries.

**Key Points:**

- Limited data is available to facilitate comprehensive radiographer workforce planning across Europe.
- A structured, workload-based approach for radiographer staffing using the World Health Organization’s Workload Indicators of Staffing Need method is proposed.
- Improved data will support workforce planning and the appropriate recognition and utilisation of emerging and advanced professional roles.

## Introduction

The EU-REST (European Union Radiation, Education, Staffing and Training) project evaluated the current medical ionising radiation workforce across the EU Member States, with radiographers being one of the core professions [1–5]. Given the critical role of radiation-based medical procedures in diagnostics and treatment, ensuring an adequately staffed and trained radiographer workforce is essential for patient and staff safety [6].

This paper focuses specifically on radiographers, a pivotal group working across medical imaging, nuclear medicine, and radiotherapy. Their role varies across EU Member States depending on national regulations and classifications. By analysing workforce distribution, educational preparedness, and training gaps, the EU-REST project highlights key areas for policy development and harmonisation [1, 4]. The findings contributed to shaping evidence-based guidelines and recommendations to strengthen radiographer education and staffing models, ultimately enhancing the safety and quality of ionising radiation applications in medical settings across the EU.

The term ‘radiographer’ encompasses three key branches of the profession recognised at the European level: medical imaging, radiotherapy, and nuclear medicine [4, 7]. The radiography profession has undergone significant transformations in response to technological advancements, evolving healthcare policies, and changing population health needs. These changes had a profound impact on radiographers’ roles, work practices, and workforce demands [7].

As the profession continues to evolve, radiographers are increasingly taking on extended and advanced roles, enhancing service efficiency, improving patient outcomes, and enabling less invasive procedures. The development of emerging technologies, particularly artificial intelligence (AI), alongside demographic shifts and disease burden, is expected to further elevate the importance of radiographers in modern healthcare [6–8].

Despite their critical role, determining radiographer workforce requirements remains a complex challenge. Unlike some other healthcare professions, there is no universally accepted methodology to define workload per radiographer, nor is there consensus on the optimal number of imaging/therapy examinations or equipment needed per population. Existing workforce guidelines are fragmented and lack standardisation, particularly concerning staffing ratios across different modalities [9].

The EU-REST project sought to address this issue by proposing evidence-based guidelines for radiographer staffing across medical imaging, nuclear medicine, and radiotherapy. The project considered radiographers’ involvement in clinical practice, teaching, research, and management, acknowledging the broad scope of their responsibilities. The findings highlighted the urgent need for a harmonised workforce planning approach to ensure sustainable staffing levels while maintaining quality and safety in medical radiation applications [5].

## Materials and methods

Radiographer staffing guidelines developed in EU-REST were formulated by a multidisciplinary group, with representatives from each relevant discipline and profession (Radiographers, Radiologists, Nuclear Medicine Physicians, Radiation Oncologists, and Medical Physics Experts) [1]. Given the significant variations in roles and responsibilities across and within disciplines, the guidelines were designed to reflect these differences while maintaining a standardised approach to staffing recommendations [5].

The guidelines were established through the EU-REST data collection, which included a comprehensive survey conducted among professional organisations, national societies, government agencies, and regulatory bodies, as well as an extensive literature review of national, EU, and international staffing guidelines. The collected data informed the determination of optimal staffing levels, considering key factors such as available equipment, expected workload, and the complexity of clinical practices [1–5].

To ensure a systematic and adaptable methodology, a baseline approach was defined to calculate the minimum staffing requirements for each profession within each discipline. This baseline served as a reference point, with additional staffing needs determined based on increased workload, complexity, equipment availability, and evolving roles and responsibilities. The methodology also allowed for projections to support future service expansion and role development, ensuring the long-term applicability of the guidelines [2, 3, 5]. This structured approach provided a robust framework for workforce planning, ensuring safe and effective staffing in medical applications involving ionising radiation [5].

## Common themes across professions

Despite variations across professions, several common themes emerged:

- Workforce availability: national registries of professionals should be established, using standardised data collection methods to track workforce demographics, subspecialties, and employment settings. Regulatory bodies should maintain these registries to monitor staffing shortages and support EU-wide data accessibility.
- Workforce planning: each profession proposed its own method for calculating staffing requirements, based on profession-specific factors.
- Quality and safety: continuing professional development (CPD) should be mandatory to maintain competence and ensure high-quality, safe medical practices.

## Existing methods for calculating radiographer numbers

Research on radiographer staffing methodologies was scarce, with few national guidelines available. The EU-REST study identified the following key findings:

- Future workforce needs: there is a pressing need to accommodate advanced practice roles, promote career progression, and enhance job satisfaction for radiographers.
- National-level guidelines: most existing staffing recommendations originate from the UK, with limited guidance available from other European countries. These sources address topics such as workforce issues, skills mix, staffing levels, service delivery models, equipment availability, and training requirements [6, 9–15].
- International perspectives: in the Netherlands, workforce planning is based on projected increases in patient numbers, with a focus on expanding existing departments rather than establishing new ones. This approach enables efficient introduction of new technologies and facilitates staff subspecialisation [16].
- Modality-specific staffing guidelines: only one source specified staffing numbers, recommending two radiographers per CT/MRI scanner and one radiographer per general X-ray or ultrasound room [9]. However, no methodology was provided to justify these figures, highlighting the need for a standardised approach.

## Workforce planning challenges and considerations

An effective radiographer workforce must be appropriately allocated to ensure optimal service delivery in terms of cost, quality, and efficiency. Key challenges include [17–21]:

- Staff attrition and burnout: retaining radiographers is crucial, particularly in addressing work-related stress and burnout.
- Health and safety considerations: workforce planning must incorporate measures to mitigate radiation exposure, musculoskeletal strain, and psychosocial risks.
- Role expansion: radiographers are increasingly involved in advanced imaging techniques, image interpretation and reporting, interventional procedures, and AI-assisted diagnostics, requiring new workforce models.
- Lack of standardised metrics: existing workforce models fail to account for local variations in service demand, evolving radiographer roles, and technological advancements.

## Recommended approach for radiographer workforce planning

To establish a practical and adaptable staffing model, the EU-REST study recommends a workload-based approach for radiographer staffing, aligned with the World Health Organization’s (WHO) Workload Indicators of Staffing Need (WISN) method [22]. This model ensures the right allocation of personnel by considering the:

- Right number of people,
- Right skills and competencies,
- Right place and time,
- Right work output and efficiency,
- Right cost-effectiveness.

Traditional staffing models, such as population-to-staff ratios (e.g., number of radiographers per 10,000 people) or staff-per-modality formulas (e.g., two radiographers per MRI scanner), fail to address local service demands, staff roles, and technological influences. The WISN approach provides a structured alternative, incorporating service statistics and workload assessments to optimise workforce planning.

## Proposed radiographer staffing framework

Building upon workload-based methodologies, Bam et al introduced a seven-step framework for radiographer staffing, integrating clinical and non-clinical activities while considering leave allowances [23]. The EU-REST study recommends the adoption of this structured approach:

### Step 1: Define workforce planning objectives

Identifying the primary factors influencing workforce demand, such as technological advancements, patient demand, and service delivery challenges [22].

### Step 2: Collect and analyse data

Gathering comprehensive data on radiographer workload, equipment utilisation, and patient demographics. Considerations include departmental scope, service complexity, training commitments, and research involvement [24].

### Step 3: Determine available working time (AWT)

Calculating actual working hours per full-time equivalent (FTE) radiographer, accounting for leave allowances (e.g., public holidays, sick leave, training, etc.). The formula used is:

$${{\rm{AWT}}}={{\rm{A}}}{-}({{\rm{B}}}+{{\rm{C}}}+{{\rm{D}}}+{{\rm{E}}})$$

where:

- A = Total working days per year
- B = Public holidays
- C = Annual leave
- D = Sick leave
- E = Other leave (e.g., training)

The formula calculates the AWT in working days per year, which can be translated to working hours per year by multiplying the AWT in working days by the number of daily working hours.

$${{\rm{AWT}}}=[{{\rm{A}}}{-}({{\rm{B}}}+{{\rm{C}}}+{{\rm{D}}}+{{\rm{E}}})] \, \times \, {{\rm{F}}}$$

where:

- F = Daily working hours

### Step 4: Develop a comprehensive task list

Identifying and categorising all clinical and non-clinical tasks performed by radiographers to determine workload distribution.

### Step 5: Assign task durations and frequency

Estimating the time required for each task and its frequency of occurrence using historical data, time-motion studies, and expert consensus [25–27].

### Step 6: Calculate workload and staffing requirements

For each imaging modality, a separate workload table is recommended to quantify the workload associated with various activities (Table 1) [28].

**Table 1:** Template for calculating workload per modality, calculated with an example for mammography

Activity It is important to identify and list each activity related to the modality, together with their associated frequency and mean time estimates	Clinical (C)/ Non-clinical (NC)	Activity frequency (AF) (per annum)	Mean time estimate for examination (MTE) (hours)	Workload (hours) = (AF × MTE)
Mammogram	C	2542.8	0.359	912.865
Monthly mammography staff meeting	NC	12	1.042	12.504
Total workload for modality (∑) (hours per annum)				925.369

The conversion of workload to theoretical FTEs, and subsequently to actual FTEs, requires several key considerations:

- Establishing a realistic application level for staff members.
- Implementing a rational approach to rounding theoretical FTE values to determine actual FTEs.
- Assessing the feasibility of pooling staff across modalities when determining actual FTEs and accounting for additional FTEs needed for leave coverage.

It is impractical to assume that staff always operate at full capacity. A practical utilisation rate should be factored into FTE calculations, with a recommended allowance of 20%, yielding a utilisation rate (U) of 80% for radiographers [24]. The theoretical FTE requirement is calculated as follows:

$${{\rm{Theoretical}}} \ {{\rm{FTE}}} = \frac{\sum {{\rm{AWT}}}} {{{\rm{A}}} \, \times \, {{\rm{U}}}} \quad {{\rm{for}}}\ {{\rm{each}}}\ {{\rm{modality}}}$$

where:

- U = the specified utilisation rate,
- A = is the total available working hours per year,
- Theoretical FTE represents the number of FTEs needed to manage the workload of a given modality at the specified utilisation rate, excluding leave considerations.

To incorporate leave allowances, the required FTEs for each modality are determined using the formula:

$${{\rm{FTE}}}={{\rm{Theoretical}}} \, {{\rm{FTE}}} \, \times \, (1+{{\rm{L}}})$$

where L represents the leave relief factor, which is calculated as:

$${{\rm{L}}}({{\rm{leave}}}\ {{\rm{relief}}}\ {{\rm{factor}}})=\frac{{{\rm{H}}}\times ({{{\rm{D}}}}_{{{\rm{p}}}}+{{{\rm{D}}}}_{{{\rm{s}}}}+{{{\rm{D}}}}_{{{\rm{a}}}}+{{{\rm{D}}}}_{{{\rm{o}}}})}{{{\rm{A}}}}$$

with:

- D_p_ = the average number of public holidays occurring on operational days,
- D_s_ = the average sick leave days per employee annually,
- D_a_ = the average annual leave days per employee,
- D_o_ = other leave categories (e.g., training, family responsibility leave),
- H = the standard daily working hours, excluding break times.

To translate theoretical FTEs into actual staffing requirements, rounding up to the nearest whole number is necessary [22]. Additionally, in settings where radiographers cannot work alone, extra staffing must be allocated accordingly (a minimum/staff staffing level must be established for individual modalities/settings and is a factor in step 4 and Step 6 above. Consensus at the national level is required to provide a uniform approach).

### Step 7: Analysis and interpretation of results

Following the framework’s implementation, an analysis should be conducted to:

- Compare current staffing levels with those calculated using the framework.
- Determine the ratio between these figures.

Only after thorough analysis and interpretation of the results can informed decisions be made regarding optimal staffing strategies.

## Workforce planning: recognising emerging essential roles

A structured methodology has been proposed for radiographers to facilitate workforce planning and ensure adaptability to evolving demands, such as increasing examination volumes and anticipated technological advancements. Effective workforce planning necessitates a comprehensive assessment of staffing requirements; however, it is widely acknowledged that over the next decade, concerted efforts will be required across all EU Member States to establish and integrate key emerging roles within the radiographer profession [7, 9, 13]. This integration must be achieved through formal role recognition, targeted education and training initiatives, and the inclusion of these roles within workforce planning models.

The following emerging roles are critical to the advancement of the radiographer profession:

- **Clinical research radiographers**: these professionals are clinical radiographers with dedicated research time, fostering innovation within their institutions and contributing to the development of clinician-scientists within the profession. Such roles may involve joint appointments with higher education institutions (HEIs) to strengthen the link between academic research and clinical practice.
- **Educators (clinically-based and higher education institution (HEI)-based)**: the recognition of clinical educators is essential for the effective training of both students and practicing professionals. These educators require additional training and protected time to support teaching responsibilities. Furthermore, within HEIs, the continuous development of the academic workforce is crucial to sustaining high educational standards and advancing radiographic practice.
- **Leadership and management**: expanding leadership and management capacity is vital for the growth of the radiographer profession. Enhancing radiographers’ roles in service delivery, patient care, and health system management presents significant opportunities to improve patient outcomes and optimise healthcare services at national and institutional levels.
- **Advanced practitioners**: across the three primary branches of the profession, medical imaging, nuclear medicine, and radiotherapy, there is substantial potential to enhance patient care, clinical outcomes, and service efficiency through the structured implementation of advanced practice roles. These roles must be supported by comprehensive education, specialised training, and adherence to evidence-based practice. It is important to note that such advanced practice roles have not yet been implemented across all EU Member States; thus, recognition and regulation vary considerably [29].
- **Quality, quality improvement, and risk management leads**: radiographers are well-positioned to assume leadership roles in quality assurance and risk management within medical imaging, nuclear medicine, and radiotherapy. In addition to broad institutional responsibilities, it is imperative that radiographers occupy specialised positions, such as radiation safety officers [30], magnetic resonance safety officers [31], and clinical audit leads [32], to ensure compliance with safety standards [33] and continuous quality improvement.

Recognising and formally integrating these essential roles into workforce planning is imperative for the sustainable growth of the radiographer profession. Addressing these workforce needs will enhance professional development, improve healthcare service delivery, and ultimately lead to better patient outcomes.

## Conclusion

The EU-REST project highlighted the urgent need for standardised workforce planning methodologies for radiographers across medical imaging, nuclear medicine, and radiotherapy. Current staffing models lack consistency and fail to address evolving service demands. To ensure the sustainability and effectiveness of the radiographer workforce across EU Member States, a coordinated and evidence-based approach to workforce planning is essential. The adoption of a workload-based methodology, alongside the implementation of a harmonised framework for workforce calculation, and publication of data at the European level, will enable a more comprehensive evaluation of radiographer workforce dynamics. Establishing national registries and structures for the systematic review of workforce data will further support strategic planning and workforce sustainability. Additionally, recognising emerging and essential roles, including advanced practice, alongside implementing structured initiatives for their development with appropriate education, training, and governance frameworks, is critical for addressing evolving healthcare demands. By prioritising these recommendations, EU Member States can enhance workforce capacity, optimise service delivery, and improve patient outcomes, ensuring the radiographer profession remains adaptable and responsive to future challenges.

## Data Availability

European Commission: European Health and Digital Executive Agency, Analysis on workforce availability, education and training needs for the quality and safety of medical applications involving ionising radiation in the EU—Status and recommendations—Final report, Publications Office of the European Union, 2025, https://data.europa.eu/doi/10.2925/2213975.

## Abbreviations

AI: Artificial intelligence
AWT: Available working time
EU: European Union
EU-REST: European Union Radiation, Education, Staffing and Training
FTE: Full-time equivalent
HEI: Higher education institution
WISN: World Health Organization’s Workload Indicators of Staffing Need
